# Ligand effect on the catalytic activity of porphyrin-protected gold clusters in the electrochemical hydrogen evolution reaction†
†Electronic supplementary information (ESI) available. See DOI: 10.1039/c7sc03997b


**DOI:** 10.1039/c7sc03997b

**Published:** 2017-11-03

**Authors:** Daichi Eguchi, Masanori Sakamoto, Toshiharu Teranishi

**Affiliations:** a Department of Chemistry , Graduate School of Science , Kyoto University , Gokasho , Uji , Kyoto 611-0011 , Japan; b Institute for Chemical Research , Kyoto University , Gokasho , Uji , Kyoto 611-0011 , Japan . Email: sakamoto@scl.kyoto-u.ac.jp ; Email: teranisi@scl.kyoto-u.ac.jp

## Abstract

The “ligand effect” can be used as a novel strategy for enhancing the catalytic properties of metal clusters. Herein, we report the ligand effect of porphyrin derivatives on gold clusters (AuCs, size <2 nm) and gold nanoparticles (AuNPs, size >2 nm) in the electrochemical hydrogen evolution reaction (HER) at pH 6.7. The current density of porphyrin face-coordinated AuCs at –0.4 V *vs.* reversible hydrogen electrode (RHE) was 460% higher than that of phenylethanethiol-protected AuCs. X-ray photoemission spectroscopy indicated that the approach of porphyrin to the Au surface induced charge migration from the porphyrin to the Au core, leading to a shift in the 5d state of AuCs that resulted in enhanced HER activities. This ligand effect is pronounced in the cluster region due to the large surface-to-volume ratio. These results pave the way for enhancing catalytic activity of metal clusters using ligand design.

## Abstract 2

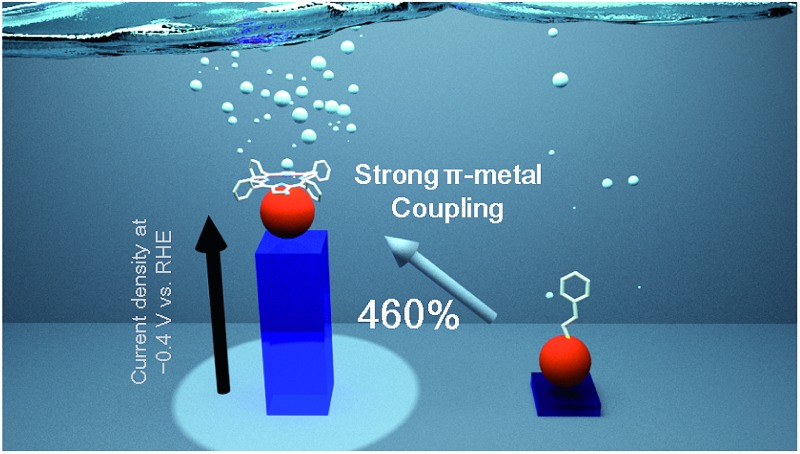
Ligand effect on the catalytic activity of gold clusters in the electrochemical hydrogen evolution reaction.

## 

Metal clusters (MCs) are promising catalysts due to their unique quantized electronic structures and large surface-to-volume ratios.^1^ Recent advances in catalytic applications of MCs have resulted from the development of precise synthetic methods for MCs, which have allowed systematic investigation of their catalytic activities in relation to their geometrical and electronic structures.^2^ Because of their large surface-to-volume ratios, the electronic structures of MCs should be perturbed by organic ligands.^3^ However, little research has been conducted regarding the ligand effect on the catalytic activity of MCs. In 2016, Jin and coworkers published a seminal report describing the ligand effect on gold clusters (AuCs, size <2 nm) in the Ullmann reaction.^4^ Aromatic thiol-protected AuCs exhibited both a higher conversion efficiency and higher product selectivity than their aliphatic thiol-protected counterparts. Moreover, Tsukuda and coworkers reported that the catalytic activity of polyvinylpyrrolidone (PVP)-protected AuCs in the aerobic oxidation of 4-hydroxybenzyl alcohol was promoted by electron transfer from PVP to the Au core.2*b* These results indicated that the role of the ligands had a significant effect on catalytic activity. Therefore, the concept of the ligand effect, which has generally been observed in metal complex catalysts as well as Au(i) complex, can also be applied to MCs.^5^

Recently, it was found that the face-on coordination of porphyrin derivatives with AuCs or gold nanoparticles (AuNPs, size >2 nm) caused a dramatic perturbation in the electronic structure of the porphyrin derivatives.^6^,^7^ These systems resembled AuNPs supported on graphene nanoribbons (GNRs), which have been shown to significantly enhance CO_2_ reduction *via* charge migration.^8^ These reports inspired us to consider whether face-coordination of porphyrin derivatives with AuCs or AuNPs could influence their catalytic activity in reduction reactions.

In this study, we systematically studied the ligand effect of porphyrin derivatives on AuCs and AuNPs of various sizes in the electrochemical hydrogen evolution reaction (HER). The HER is a key catalytic reaction for producing clean energy from inexhaustible water. Gold is a chemically stable metal element, even in the cluster region.2*a* Herein, we show that the ligand effect of porphyrin derivatives significantly improves the HER activity of AuCs. AuCs with face-coordinated porphyrin derivatives at –0.4 V *vs.* reversible hydrogen electrode (RHE) show a 460% higher current density and negatively shifted overpotential (70 mV) compared with phenylethanethiol-protected AuCs. The dramatic catalytic enhancement in the cluster region is attributed to charge migration from the porphyrin to the Au core. This ligand effect provides a novel strategy for enhancing the catalytic activity of MCs.

To elucidate the ligand effect on the catalytic activity of AuCs, we designed and synthesized SC_*n*_P (*n* = 1,2) using a previously described method (Fig. 1 and 2).6*b* The distance and electronic interactions between the porphyrin ring and Au surface can be tuned by changing the number of methylene groups in SC_*n*_P. Single-crystal X-ray diffraction analysis of SC_*n*_P (*n* = 1,2) showed that the distances between sulfur atoms and the porphyrin ring were 3.4 and 4.9 Å for SC_1_P and SC_2_P, respectively (Fig. 2). Acetylthio groups, which served as binding sites for the Au surface, faced in the same direction toward porphyrin ring, resulting in face-on coordination of SC_*n*_P on the Au surface. SC_*n*_P-protected AuCs and AuNPs (SC_*n*_P/AuCs and SC_*n*_P/AuNPs) were synthesized by reducing the Au precursor in the presence of SC_*n*_P (see ESI† for detailed synthetic procedures). The obtained crude products were purified by GPC to remove byproducts and unreacted free SC_*n*_P. Fig. S1a† shows the chromatograms of crude SC_*n*_P/AuCs. Major components in the chromatograms were collected for further characterization using TEM, MALDI-TOF MS, ICP-AES, and UV-vis absorption spectroscopy. As the retention times of both SC_*n*_P/AuCs were similar, their sizes should be identical (Fig. S1b†). Cluster sizes and size distributions were analysed using TEM (Fig. 2a and d). The size distributions shown in Fig. S2† were obtained by measuring 500 AuCs. The sizes of both SC_*n*_P/AuCs were estimated to be 1.3 ± 0.2 nm. The chemical compositions of the SC_*n*_P/AuCs were determined using MALDI-TOF MS in linear positive mode and ICP-AES. Peaks observed at approximately 22.9 and 26.0 kDa in the MALDI-TOF MS spectra were assigned to structures consisting of 77 Au atoms and 8 SC_1_P molecules (SC_1_P/AuC), and 75 Au atoms and 11 SC_2_P molecules (SC_2_P/AuC), respectively (Fig. S3†). Au/sulfur atomic ratios determined by ICP-AES were 77 : 32 and 75 : 44 for SC_1_P/AuC and SC_2_P/AuC, respectively, indicating that 8 SC_1_P molecules and 11 SC_2_P molecules were attached to single AuC. These ICP-AES results were in good agreement with the MALDI-TOF MS results. By considering the occupied area of a porphyrin molecule (1.2 nm^2^) and distances between the acetylthio groups and porphyrin ring, approximately 10 SC_1_P molecules and 13 SC_2_P molecules were determined to cover single AuC (Fig. S4 and Table S1†). Therefore, we concluded that the obtained AuCs were protected by SC_*n*_P in a face-coordinated fashion, as previously reported.^6^,^9^ SC_*n*_P/AuNPs of 2.2 or 3.8 nm in size were synthesized by the reduction of Au precursor at –50 or 0 °C, respectively (Fig. 2b, c, e, and f). SC_*n*_P/AuNPs were characterized using similar procedures to those used for SC_*n*_P/AuCs. The number of SC_*n*_Ps coordinated with the AuNPs was estimated by ICP-AES and the calculated average number of Au atoms in a single NP is summarized in Table S1.† 10

**Fig. 1:**
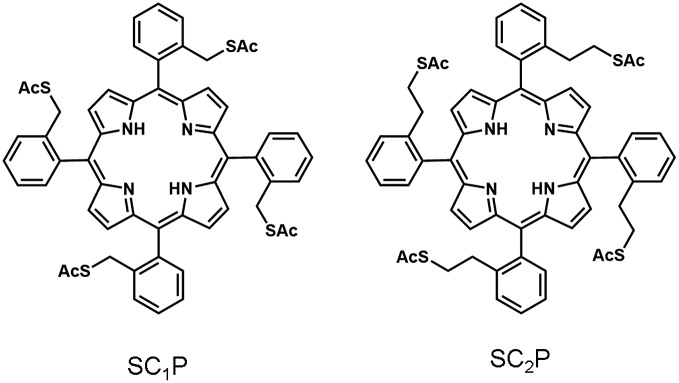
Chemical structures of SC_1_P and SC_2_P.

**Fig. 2:**
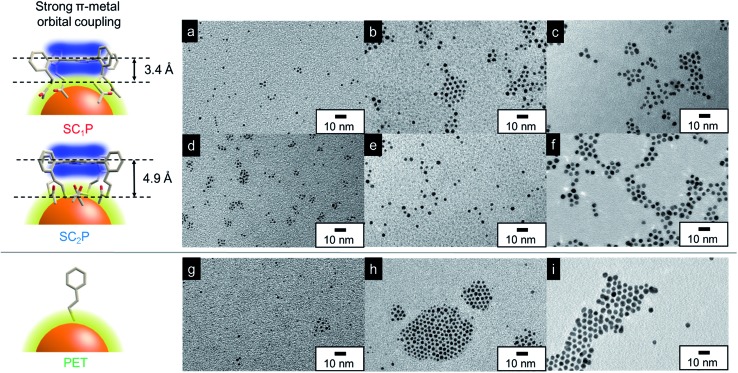
Schematic illustration of coordination fashions of SC_*n*_P and PET on Au surface. Chemical structures of SC_*n*_P were obtained by single-crystal X-ray diffraction analyses.6*b* TEM images of (a) 1.3 nm-, (b) 2.2 nm-, (c) 3.8 nm-SC_1_P/AuCs or AuNPs, (d) 1.3 nm-, (e) 2.2 nm-, (f) 3.8 nm-SC_2_P/AuCs or AuNPs, (g) 1.2 nm-, (h) 2.3 nm-, and (i) 3.8 nm-PET/AuCs or AuNPs.

Face-coordination of the porphyrin derivatives on AuCs was also confirmed by the optical properties. We previously reported systematic studies of interactions between face-coordinated porphyrin derivatives and AuCs and AuNPs with respect to optical properties.^6^ Face-coordination of porphyrin derivatives on AuCs caused a bathochromic shift and spectral broadening due to π-metal orbital coupling. Fig. 3a and b show absorption spectra of SC_*n*_P/AuCs and free SC_*n*_P in DMF at room temperature. The molar concentrations of SC_*n*_Ps on AuCs were estimated using ICP-AES. The peak positions of Soret bands for SC_1_P and SC_1_P/AuCs were 420 and 425 nm, while those of SC_2_P and SC_2_P/AuCs were 418 and 422 nm, respectively (Table 1). Moreover, damping of the molar absorption coefficients was observed. The damping ratios of SC_1_P and SC_2_P on AuCs were 28% (from 3.9 × 10^5^ to 1.1 × 10^5^ M^–1^ cm^–1^) and 54% (from 3.9 × 10^5^ to 2.1 × 10^5^ M^–1^ cm^–1^), respectively. Considering the differences in the number of methylene groups in SC_*n*_P, the bathochromic shifts, damping of the molar absorption coefficients, and peak broadening was strongly dependent on the distance between the porphyrin ring and the AuC surface. Notable damping of the molar absorption coefficients and peak broadening was also observed for SC_*n*_P-coordinated AuNPs (Fig. S8 and Table S2†). As the density of state (DOS) for AuNPs is higher than that of AuCs, π-metal orbital coupling would be favorable in SC_*n*_P/AuNPs. These results indicated that the SC_*n*_P ligands were coordinated with the AuCs and AuNPs in a face-on fashion.

**Fig. 3:**
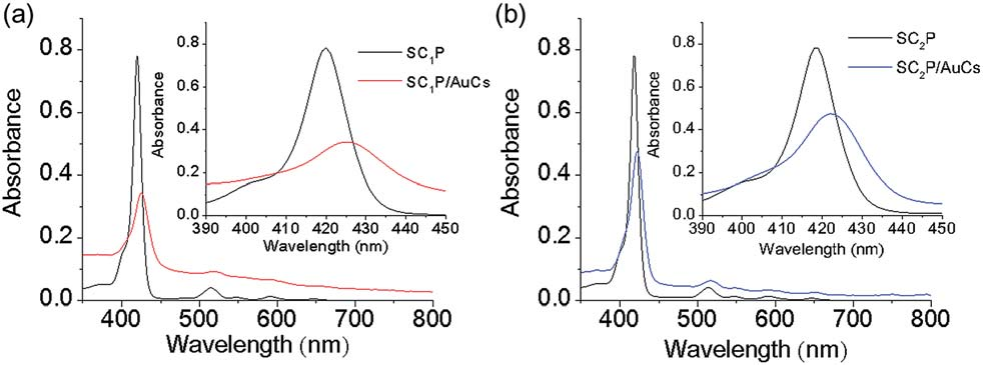
UV-vis absorption spectra of (a) SC_1_P (black) and SC_1_P/AuCs (red) and (b) SC_2_P (black) and SC_2_P/AuCs (blue) in DMF. Molar concentrations were 2.0 μM for free SC_*n*_P and SC_*n*_P on AuCs. Molar concentrations of SC_*n*_P/AuCs were estimated using ICP-AES. Insets show magnified spectra at 390–450 nm.

**Table 1:** Optical properties of SC_*n*_P/AuCs and free SC_*n*_P in DMF

Compounds	*λ*_max_ (nm)	*ε* ^*a*^ (× 10^5^ M^–1^ cm^–1^)	FWHM^*b*^ (nm)
SC_1_P	420	3.9	13
SC_1_P/AuCs	425	1.1	21
SC_2_P	418	3.9	12
SC_2_P/AuCs	422	2.1	19

^*a*^Molar absorption coefficient of Soret band of SC_*n*_P.

^*b*^Full width half maximum at Soret band.

The HER activities of the AuCs and AuNPs were measured in potassium phosphate buffer solution using linear sweep voltammetry (LSV). All potentials reported in this work were referenced to the RHE. The working electrodes were prepared by drop-casting the samples solutions on carbon tapes while keeping the amount of Au atoms constant. As control experiments, the HER activities of AuCs and AuNPs of various sizes protected with phenylethanethiol (PET), a conventional aliphatic thiol ligand, were investigated (see ESI† for detailed synthetic procedures; Fig. 2g–i). As shown in Fig. S9 and S10,† 2.3 nm PET/AuNPs exhibited the lowest overpotential of 10 mA cm^–2^, and the highest current density of –0.4 V. The size dependence of HER activity can be explained by the size-dependent coverage of PET molecules on the Au surface and the surface-to-volume ratio. For small AuCs, a high surface coverage of PET impedes the accessibility of water molecules to the Au surface.^11^ Although the surface coverage of PET on AuNPs decreased with increasing NP size, the surface-to-volume ratio also decreased. Consequently, 2.3 nm PET/AuNPs showed the highest HER activity among the PET/AuCs and PET/AuNPs studied. Next, we examined the ligand effect on the HER activities of SC_*n*_P/AuCs. The SC_1_P/AuCs showed an overpotential of 0.47 V for the HER, which was slightly more negative than that of SC_2_P/AuCs (Fig. 4a and Table S3†). The current densities at potentials of –0.2, –0.3, and –0.4 V for the SC_1_P/AuCs were 101, 230, and 330% higher than those of SC_2_P/AuCs (Fig. 4b). Furthermore, the current density at –0.4 V for SC_1_P/AuCs was 460% higher than that of the PET/AuCs. Since the surface coverage of the ligands in both PET/AuCs and SC_*n*_P/AuCs are almost fully covered, the surface coverage does not affect their current densities (see ESI†). These results indicated that the HER activities of the AuCs were enhanced by porphyrin coordination. This ligand effect greatly depended on the distance between the porphyrin ring and the Au surface. To exclude the possibility that species other than the SC_*n*_P/AuCs were operating as HER catalysts, control experiments were carried out using free SC_*n*_P and bare carbon tape. Under the same experimental conditions, these materials showed a minimal current response, indicating that the SC_*n*_P/AuCs catalyzed the HER (Fig. 4a). The absorption spectra of SC_*n*_P/AuCs were not changed even after the HER experiment (Fig. S11†). This result strongly evidences that the SC_*n*_P/AuCs were highly stable against HER and the enhancement of HER activity was derived from the ligand effect. Furthermore, Fig. S12† indicates that H_2_O molecules can access the Au surface through the gap of porphyrin ligands on AuCs.

**Fig. 4:**
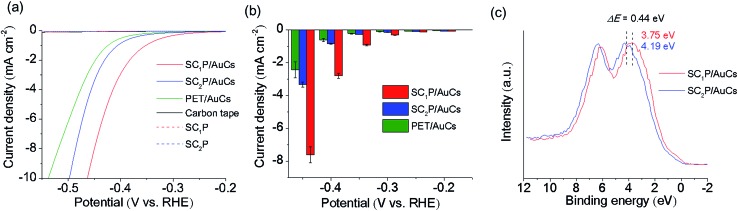
(a) HER polarization curves of SC_*n*_P/AuCs, SC_*n*_P, and bare carbon tape in 0.5 M phosphate buffer solution (pH 6.7). (b) Comparison of current densities for SC_*n*_P/AuCs and PET/AuCs at different potentials. Error bars indicate the standard deviations calculated from three runs with freshly deposited sample. (c) Valence band XPS spectra of SC_*n*_P/AuCs.

To elucidate this ligand effect, X-ray photoelectron spectroscopy (XPS) measurements of the SC_*n*_P/AuCs were carried out (Fig. 4c and S13†). The Au 5d_5/2_ core level peaks of SC_1_P/AuCs and SC_2_P/AuCs appeared at 3.75 and 4.19 eV, respectively, indicating that the approach of the porphyrin rings to the Au surface induced a shift to a lower binding energy, showing that the AuCs were electron rich. A similar phenomenon has been observed in graphene-supported AuNPs,^12^ where the charge migration from graphene to AuNPs induced a shift to a lower Au binding energy. The correlation between HER activity and Au binding energy shift can be explained by the well-recognized d-band model.^13^ When a hydrogen atom is adsorbed on a metal surface, M–H* bond formation (H* designates a hydrogen atom chemically adsorbed on a metal surface) generates new electronic structures (bonding and antibonding states). Generally, the occupancy of the antibonding state determines the M–H* bond strength. If the antibonding state is filled, the M–H* bond strength is weaker due to repulsive interactions. As the Au–H* bond strength was weak, filling the antibonding state was unfavorable for the HER. In contrast, a decrease in occupancy of the antibonding state is favorable for HER activity because the partial emptiness of the antibonding state makes the M–H* bond strong.13*c* The coordination of porphyrin molecules with the Au core shifted the 5d state of Au due to charge migration. This charge migration induced the shift of the 5d state to the Fermi level, which made the antibonding state partially empty. As a result, the coordination of porphyrin enhanced the HER activity of the AuCs.

Size-dependent HER activity was also observed in the SC_*n*_P/AuNPs. The trend in size-dependent overpotential for SC_*n*_P/AuNPs was similar to that of PET/AuNPs, exhibiting a volcano relationship (Fig. 5a, S14 and Table S3†). The 2.2 nm SC_1_P/AuNPs showed the lowest overpotential and highest current density at –0.4 V in the present work. Notably, the ligand effect was larger in smaller materials. The current density of the AuCs at –0.4 V was enhanced by 460% when changing the ligand from PET to SC_1_P, whereas that of the 3.8 nm AuNPs was enhanced by only 106% (Fig. 5b and c). This result showed that the ligand effect of porphyrin was notable in the cluster region because of their large surface-to-volume ratio.

**Fig. 5:**
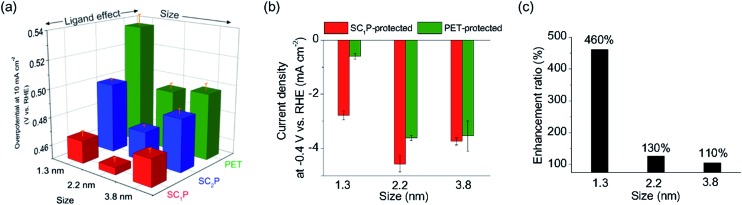
(a) Comparison of overpotential at –10 mA cm^–2^ for SC*_n_*P-and PET-protected AuCs and AuNPs. (b) Comaparison of current density at –0.4 V *vs.* RHE for SC_1_P- and PET- protected AuCs and AuNPs. Error bars indicate standard deviations obtained from experiments using freshly deposited samples. (c) Enhancement ratio of current density by ligand effect of porphyrin. Enhancement ratio = current densities at –0.4 V *vs.* RHE of SC_1_P-protected AuCs and AuNPs/current densities of PET-protected ones.

In conclusion, we have demonstrated a novel strategy for enhancing the electrochemical HER activity of AuCs. The face-on coordination of porphyrin derivatives with AuCs significantly promoted their HER activity in comparison with PET/AuCs. The configuration and electronic properties of ligands on AuCs are important factors in determining their catalytic activities. This ligand effect is dramatic in the cluster region due to the large surface-to-volume ratio, and could be applied to other metal nanocatalysts, such as platinum and palladium. The concept of controlling the catalytic activity of MCs using ligands could be used as a fourth parameter for controlling catalytic activity, in addition to the number of atoms (size), shape, and chemical composition.

## Conflicts of interest

The authors declare no competing financial interest.

## Supplementary Material

Supplementary information
