# Supplementary material for: Ligand effect on the catalytic activity of porphyrin-protected gold clusters in the electrochemical hydrogen evolution reaction
Source: Chem Sci. 2017 Nov 3;9(1):261–5. doi: 10.1039/c7sc03997b (PMC5869313; doi:10.1039/c7sc03997b)
Supplement: Supplementary file 1 [file SC-009-C7SC03997B-s001.pdf]

Electronic Supplementary Material (ESI) for Chemical Science.
This journal is © The Royal Society of Chemistry 2017

Supporting Information

Ligand effect on the catalytic activity of porphyrin-protected gold clusters in the electrochemical hydrogen evolution reaction

Daichi Eguchi,[a] Masanori Sakamoto,[b*] Toshiharu Teranishi[b*]

[a]Department of Chemistry, Graduate School of Science, Kyoto University, Gokasho, Uji, Kyoto 611-0011, Japan

[b]Institute for Chemical Research, Kyoto University, Gokasho, Uji, Kyoto 611-0011, Japan

1. Chemicals

All reagents were used as received without further purification. Tetrakis-5α,10α,15α,20α-(2-acetylthiomethylphenyl)porphyrin (SC_1P) was synthesized from terephthalaldehydic acid in five steps.[1] Tetrakis-5α,10α,15α,20α-(2-acetylthioethylphenyl)porphyrin (SC_2P) was synthesized from 3,4-dihydro-1*H*-2-benzopyran in three steps.[1]

2. Characterization

Low temperatures (–80 or –50 °C) were maintained using a liquid nitrogen–methanol cooling bath. Matrix-assisted laser desorption ionization–time of flight mass spectrometry (MALDI-TOF MS) was performed on a Bruker Autoflex Speed system using *trans*-2-[3-(4-*tert*-butylphenyl)-2-methyl-2-propenylidene]malononitrile (DCTB) as a matrix. The cluster-to-matrix molar ratio was set to 1:100. Gel permeation chromatography (GPC) was carried out on a BIO RAD sx-1 system, using toluene or *N*,*N*-dimethylformamide (DMF) as the mobile phase. Gel permeation chromatography–high performance liquid chromatography (GPC–HPLC) was performed on a LC-9225 NEXT system (Japan Analytical Industry Co., Ltd.) using a JAIGEL-W253 column. A DMF solution of lithium bromide (50 mM) was used as the mobile phase at a flow rate of 3.8 mL min^{-1}. Chromatograms were collected by monitoring at a wavelength of 435 nm. Inductively coupled plasma–atomic emission spectroscopy (ICP–AES) was performed on a Shimadzu ICPE-9800 system. UV-vis absorption spectra were measured at room temperature on a Hitachi U-4100 spectrophotometer. Fluorescence spectra were recorded at room temperature on a Horiba Fluorolog-3. Transmission electron microscopy (TEM) was performed using a JEOL JEM-100 microscope operated at an acceleration voltage of 100 kV. Histograms of the core diameters for the AuCs and AuNPs were constructed by measuring the sizes of 500 particles. X-ray photoemission spectroscopy (XPS) was performed using a PHI Quantum 2000 spectrometer with an Al Kα radiation source. XPS spectra were calibrated against the C 1s peak at 284.8 eV.

3. Electrochemical measurements

Electrochemical measurements were performed on an ALS611 electrochemical workstation with a standard three electrode cell. An Ag/AgCl electrode and a platinum wire were used as the reference electrode and counter electrode, respectively. Working electrodes were prepared as follows: a DMF or toluene solution of ligand-protected AuCs and AuNPs (1 mM of Au atoms, 30 μL) was dropped onto carbon tape (Toray, TGP-H-090, 1 × 3 cm^2). Carbon tape containing sample was dried in vacuo overnight. Linear sweep voltammetry (LSV), beginning at 0.1 V and ending at –0.8 V vs. reversible hydrogen electrode (RHE) with a scan rate of 100 mV s^{-1}, was conducted in potassium phosphate buffer solution (0.5 M, pH 6.7). Argon gas was bubbled through the electrolyte vigorously for 15 min to remove oxygen in the solution. The measured potentials vs. Ag/AgCl were converted to the RHE. The *iR*-drops by the solution were also corrected using the following equation:

$$E \text{ (vs. RHE)} = E \text{ (vs. Ag/AgCl)} + 0.242 + 0.059 \times \text{pH} - iR_{\Omega}$$

The error bars indicate the standard deviations from three runs using freshly deposited samples. All LSV curves in this work were obtained with the first scan.

4. Experimental Section

Synthesis of SC$_n$P-protected AuCs (SC$_n$P/AuCs)

An aqueous solution of hydrogen tetrachloroaurate (III) tetrahydrate ($HAuCl_4 \cdot 4H_2O$; 5 mL, 29.9 μmol) was added to a CH_2Cl_2 solution of tetraoctylammonium bromide (TOABr; 5 mL, 34.7 μmol), and the mixture was stirred vigorously for 10 min to transfer Au(III) ions into the organic phase. After removal of the aqueous phase, a mixture of SC$_n$P (14.7 μmol) in CH_2Cl_2 (25 mL) and methanol (30 mL) was added to the CH_2Cl_2 solution of Au(III). The mixture was stirred and cooled at –80 °C for 30 min. A solution of sodium borohydride ($NaBH_4$, 305 μmol) in methanol (1 mL) was added to the CH_2Cl_2 solution of Au(III) and SC$_n$P, and stirred at room temperature for 3 h to obtain AuCs. After removal of the solvent in vacuo, the residue was washed with methanol and collected by filtration. The crude product was purified by GPC–HPLC to obtain the SC$_n$P/AuCs. Chemical compositions of the obtained SC$_n$P/AuCs were different from those in our previous work.[1] The Au(III) reduction temperature was slightly higher than in previous work. Therefore, the growing rate of these cores slightly became faster.

Synthesis of SC$_n$P-protected 2.2-nm AuNPs (2.2-nm SC$_n$P/AuNPs)

Solutions of $HAuCl_4 \cdot 4H_2O$ (14.3 μmol) in methanol (7.5 mL) and SC$_n$P (2.79 μmol) in DMF (7.5 mL) were combined and stirred at –50 °C for 30 min. A solution of $NaBH_4$ (161 μmol) in methanol (1 mL) was then added to the mixture of Au(III) and SC$_n$P, and stirred at –50 °C for 30 min and room temperature for 30 min to obtain AuNPs. The reaction solution was then centrifuged with methanol (75 mL) and the resultant precipitate was redispersed in DMF and purified by GPC–HPLC to obtain 2.2-nm SC$_n$P/AuNPs.

Synthesis of SC$_n$P-protected 3.8-nm AuNPs (3.8-nm SC$_n$P/AuNPs)

A solution of $HAuCl_4 \cdot 4H_2O$ (28.2 μmol) and SC$_n$P (5.37 μmol) in DMF (15mL) was stirred at 0 °C for 30 min, before adding a solution of $NaBH_4$ (267 μmol) in methanol (1 mL). This solution was stirred at 0 °C for 30 min and room temperature for 30 min to obtain AuNPs. The reaction solution was centrifuged with methanol (75 mL) and the resultant precipitate was redispersed in DMF and purified by GPC to obtain 3.8-nm SC$_n$P/AuNPs.

Synthesis of phenylethanethiol (PET)-protected AuCs (PET/AuCs)

An aqueous solution of $HAuCl_4 \cdot 4H_2O$ (2.5 mL, 39.6 mg, 96.0 μmol) was added to a solution of TOABr (63.3 mg, 116 μmol) in CH_2Cl_2 (20 mL) and stirred vigorously for 10 min to transfer Au(III) ions to the organic phase. After removal of the aqueous phase, a solution of phenylethanethiol (PET, 49.7 μL, 390 μmol) in CH_2Cl_2 (128 mL) and methanol (30 mL) was added. The mixture was stirred at room temperature for 90 min and cooled at –80 °C for 30 min. A solution of $NaBH_4$ (35.4 mg, 930 μmol) in methanol (2 mL) was added, and the solution was stirred at room temperature for 1 h to obtain AuCs. After removal of the solvent in vacuo, the residue was washed with methanol and collected by filtration. The crude product was purified by GPC–HPLC to obtain PET/AuCs.

Synthesis of PET-protected 2.3-nm AuNPs (2.3-nm PET/AuNPs)

A solution of chlorotriphenylphosphine gold(I) (50.1 mg, 101 μmol) and PET (26.8 μL, 200 μmol) in $CHCl_3$ (10 mL) was stirred at 65 °C for 15 min. A solution of *tert*-butylamine borane complex (86.5 mg, 1.01 mmol) in $CHCl_3$ (1 mL) was then added and the resultant mixture was stirred at 65 °C for 9 h. After removal of the solvent in vacuo, the residue was washed with methanol and collected by filtration. Finally, the resultant precipitate was redispersed in toluene.

Synthesis of PET-protected 3.8-nm AuNPs (3.8-nm PET/AuCs)

This synthetic procedure was slightly modified from previous work.[2] Citrate-protected 3.8-nm AuNPs (CT/AuNPs) were prepared according to the literature.[3] An aqueous solution (24 μmol of Au atoms, 100 mL) of CT/AuNPs was added to a mixed solution of TOABr (54.4 mg, 99.5 μmol) and PET (134 μL, 1.00 mmol) in toluene and acetone (35 mL, 6:1 v/v) and stirred at room temperature for 30 min. After removal of the aqueous solution by syringe, the organic phase was evaporated in vacuo and the residue was washed with methanol. Finally, the residue was redispersed in toluene.

Synthesis of PET-protected 7.3-nm and 14.8-nm AuNPs (7.3-nm PET/AuNPs and 14.8-nm PET/AuNPs)

These synthetic procedures were slightly modified from previous work.[4] The 7.3-nm and 14.8-nm CT/AuNPs were prepared according to the literature.[3] An aqueous solution of CT/AuNPs (12 μmol of Au atoms, 50 mL) was added to a mixture of toluene and oleylamine (22 mL, 10:1 v/v), and stirred at 100 °C for 1 h. After removal of the aqueous solution by syringe, the organic phase was evaporated and the resulting mixture was centrifuged with ethanol (80 mL). Finally, the resultant precipitate was redispersed in toluene to obtain oleylamine-protected AuNPs (7.3-nm OAm/AuNPs and 14.8-nm OAm/AuNPs). A solution of OAm/AuNPs (1 mM of Au atoms) in toluene (30 μL) was dropped on carbon tape ($1 \times 3\ cm^2$) and dried in vacuo overnight. Carbon tape with AuNPs was dipped into a solution of PET (436 μL, 3.0 μmol) in ethanol (20 mL) at room temperature for 3 h. The carbon tape was then washed with ethanol and used as a working electrode.

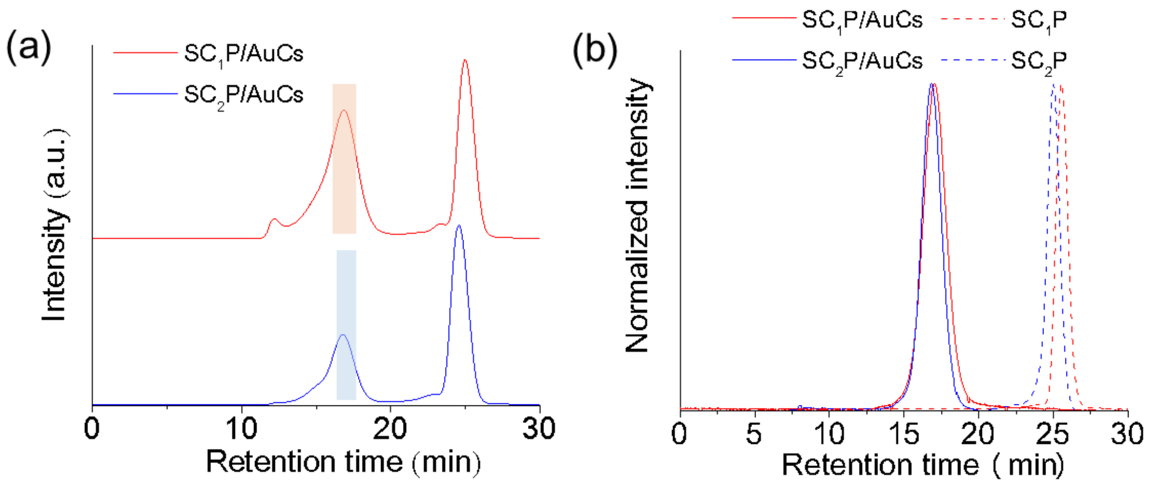

Figure S1. GPC–HPLC chromatograms of crude products SC_1P/AuCs (red) and SC_2P/AuCs (blue). Fractions were collected for further characterization.

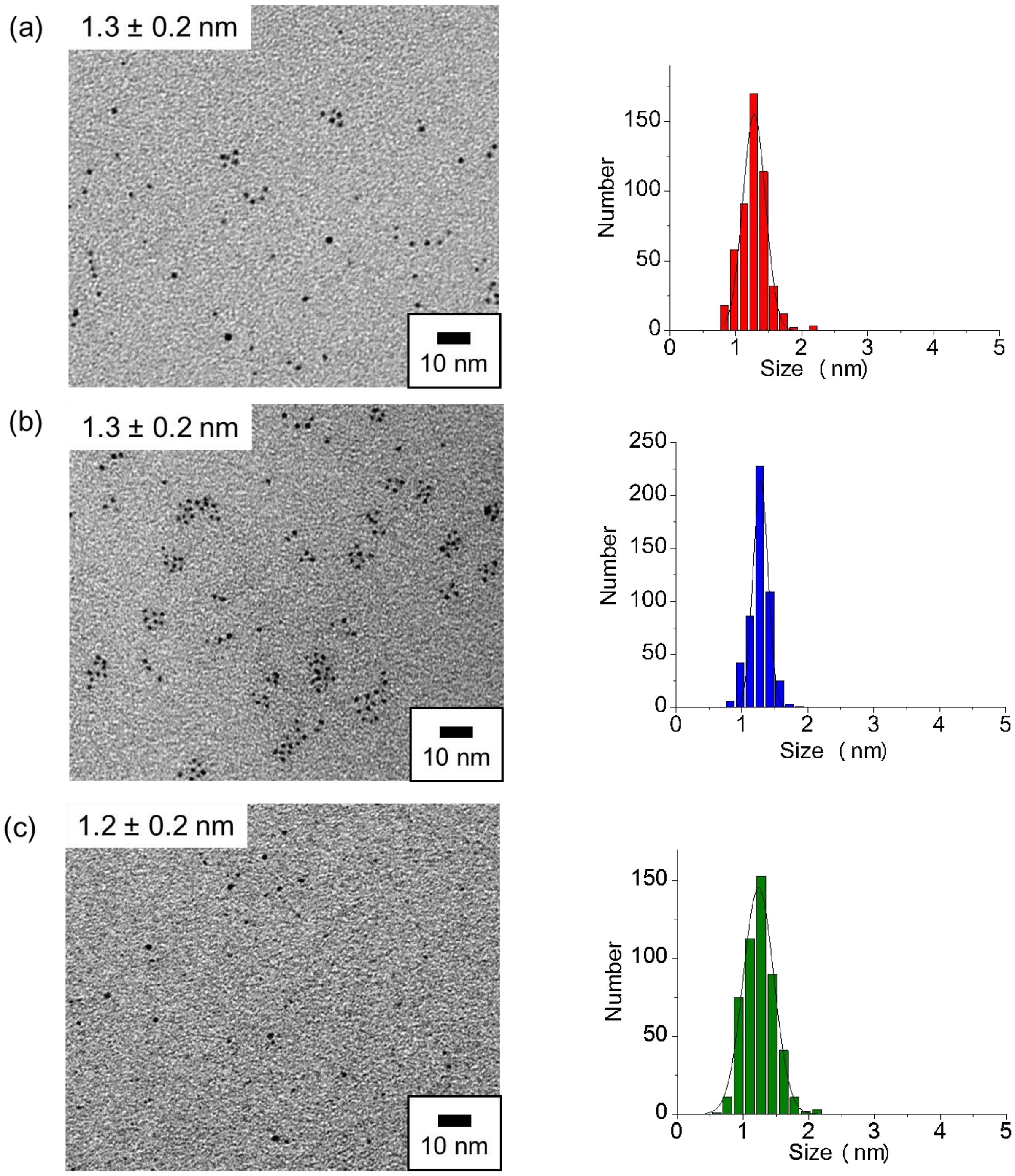

Figure S2. TEM images (left) and corresponding histograms (right) representing the size distributions of (a) 1.3 nm-SC_1P/AuCs, (b) 1.3-nm SC_2P/AuCs, and (c) 1.2-nm PET/AuCs.

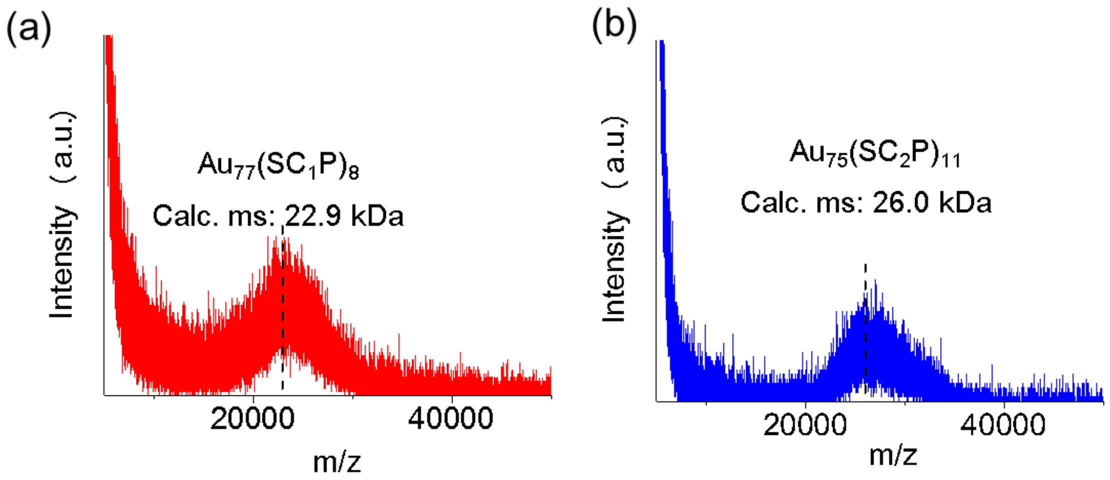

Figure S3. MALDI–TOF MS spectra of (a) SC_1P/AuCs and (b) SC_2P/AuCs in linear positive mode.

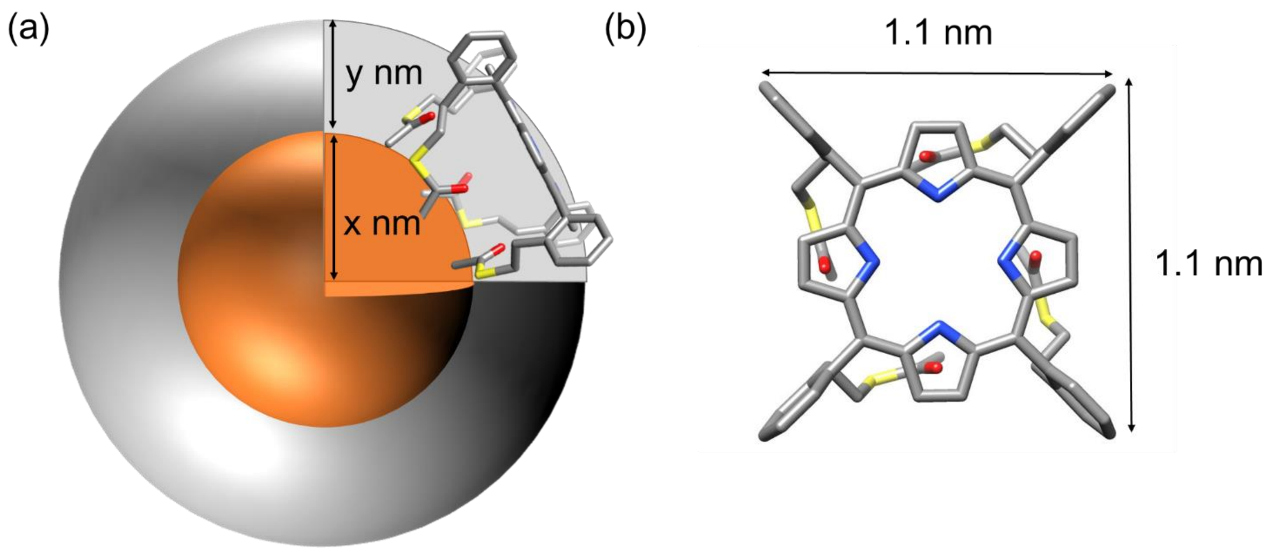

Figure S4. (a) Schematic illustration of SC_2P coordination with AuCs. (b) Structure of SC_2P from top view.[1]

Table S1. Number of SC_nP ligands coordinated on AuCs and AuNPs.

Compounds	Radius (x in Figure S4, nm)	Distance between sulfur and porphyrin ring (y in Figure S4, nm)[a]	Coordination number of SC_nP on Au surface (Estimation)[b]	Coordination number of SC_nP on Au surface (Experimental value)
SC_1P/AuCs	0.65	0.34	10	8[c]
2.2 nm SC_1P/AuNPs	1.1	0.34	21	25[d]
3.8 nm SC_1P/AuNPs	1.9	0.34	52	57[d]
SC_2P/AuCs	0.65	0.49	13	11[c]
2.2 nm SC_2P/AuNPs	1.1	0.49	26	36[d]
3.8 nm SC_2P/AuNPs	1.9	0.49	59	71[d]

[a]Estimation from single X-ray crystal structures.[1]

[b]Values were estimated using the following equation:

$(4 \times \pi \times (\text{Radius (nm)} + \text{Distance between sulfur and porphyrin ring (nm)})^2)/(\text{occupied area of porphyrin derivative } (1.2\ \text{nm}^2))$

[c]Estimated from ICP–AES and MALDI–TOF MS.

[d]Values were estimated from ICP–AES and calculated average number of Au atoms in NP. Au:S molar ratio was investigated using ICP–AES measurements and used to determine the Au:SC_nP molar ratio given that SC_nP contains four sulfur atoms in acetylthio groups. Average number of Au atoms in NP was calculated according to previous literature.[5] Number of SC_nP on the Au surface was estimated from the ratio and calculated average number of Au atoms in NP. For example, for 2.2-nm SC_1P/AuNPs, the calculated average number of Au atoms was 329, and the Au:SC_1P molar ratio was 329:25, giving the number of coordinated SC_1P on the Au surface as 25.

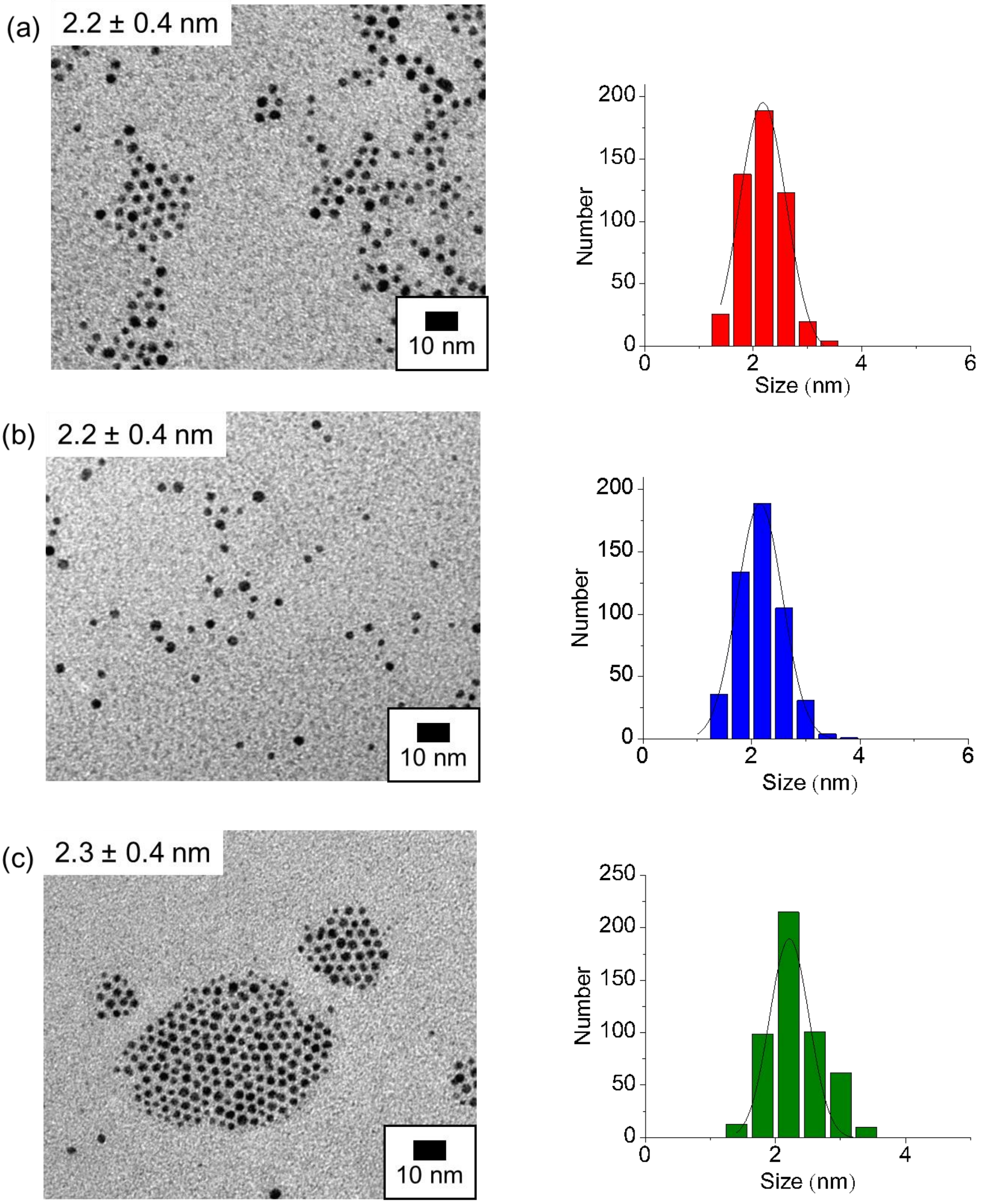

Figure S5. TEM images (left) and corresponding histograms (right) representing the size distributions of (a) 2.2-nm SC_1P/AuNPs, (b) 2.2-nm SC_2P/AuNPs, and (c) 2.3-nm PET/AuNPs.

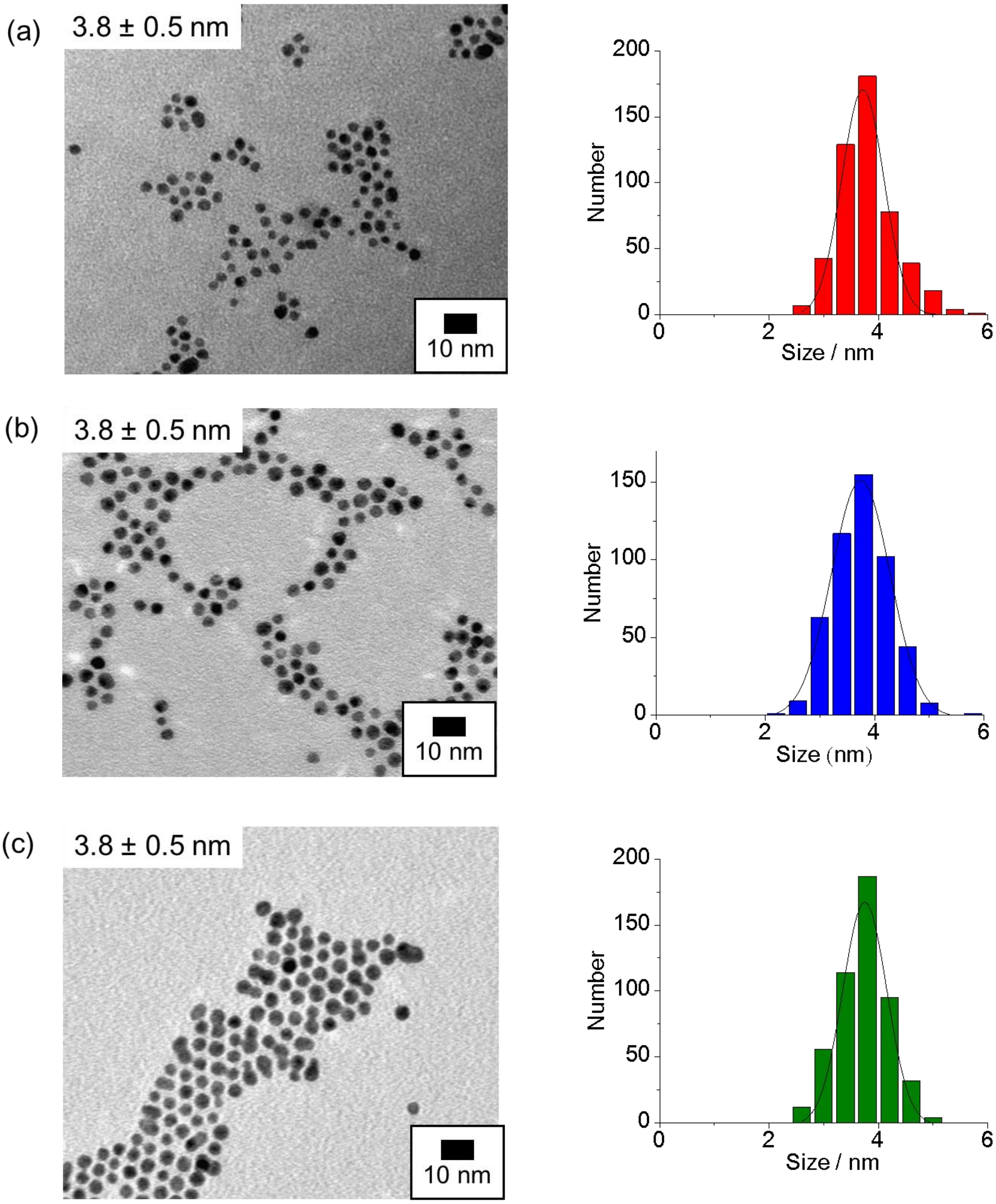

Figure S6. TEM images (left) and corresponding histograms (right) representing the size distribution of (a) 3.8-nm SC_1P/AuNPs, (b) 3.8-nm SC_2P/AuNPs, and (c) 3.8-nm PET/AuNPs.

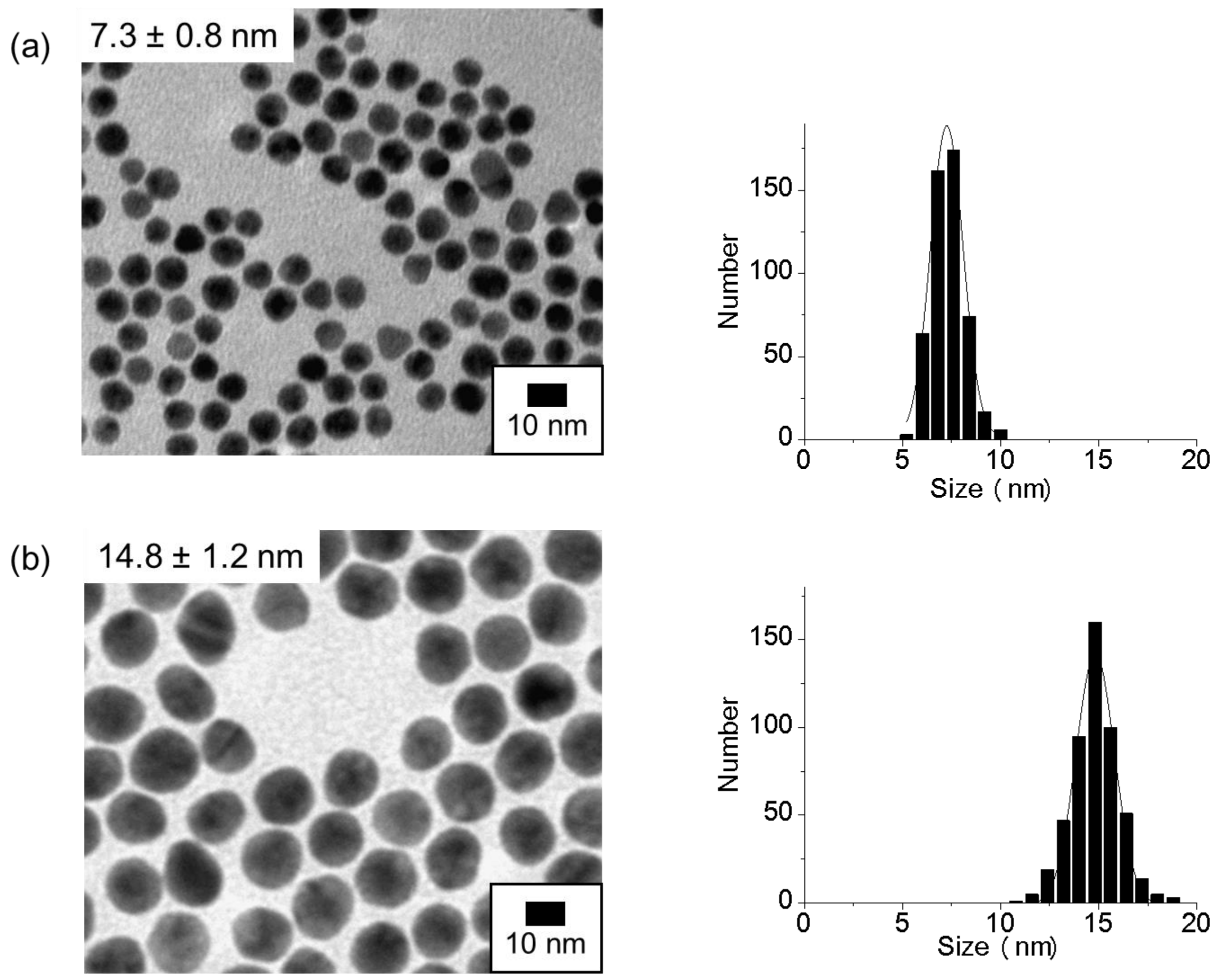

Figure S7. TEM images (left) and corresponding histograms (right) representing the size distributions of (a) 7.3-nm OAm/AuNPs, and (b) 14.8-nm OAm/AuNPs.

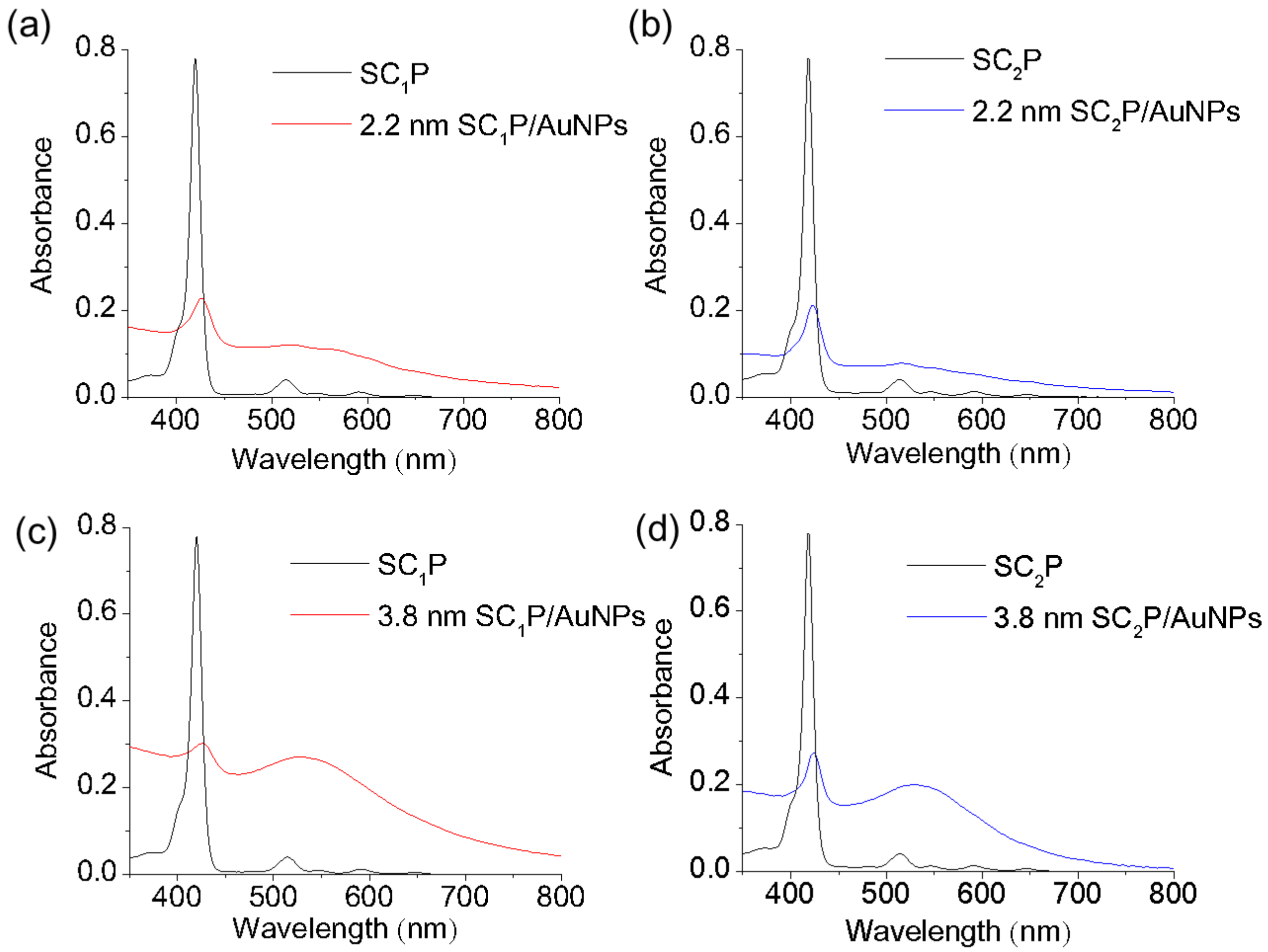

Figure S8. Absorption spectra of free SC$_n$P with (a,b) 2.2-nm SC$_n$P/AuNPs and (c,d) 3.8-nm SC$_n$P/AuNPs in DMF solution. Molar concentrations of SC$_n$P and SC$_n$P on AuNPs were 2.0 μM.

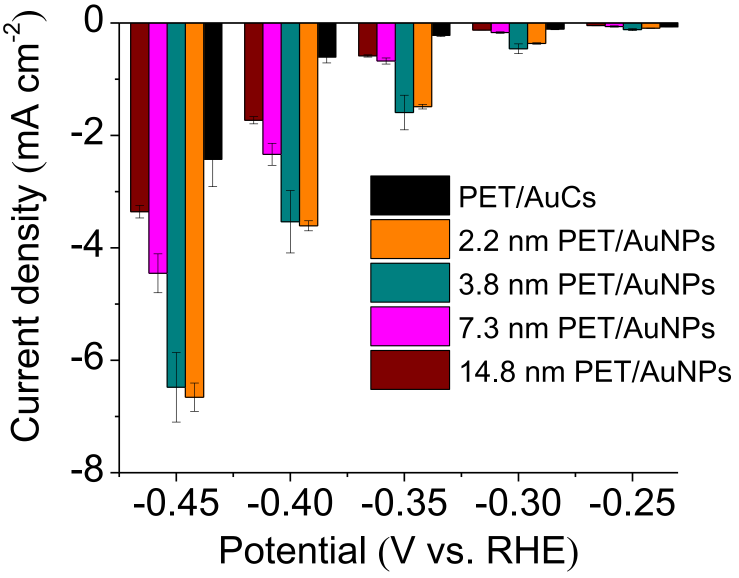

Figure S9. Comparison of current densities for PET-protected AuCs and AuNPs of various sizes at different potentials. Error bars indicate standard deviations from three runs with freshly deposited samples.

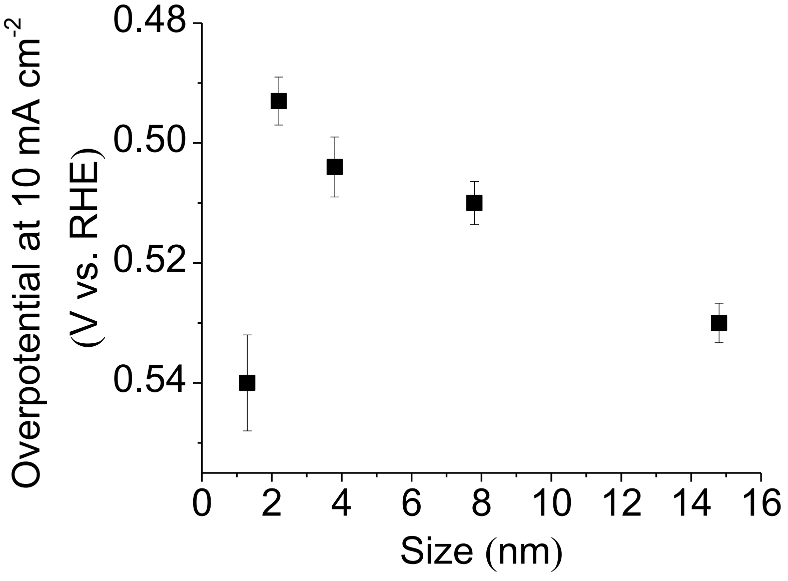

Figure S10. Overpotentials for PET-protected AuCs and AuNPs of various sizes at 10 mA cm^{-2}. Error bars indicate standard deviations from three runs with freshly deposited samples.

Estimation of the PET coverage on AuC for comparison with that of SC$_n$P/AuCs

The surface area of AuCs (d = 1.2 nm) was calculated as follows,

$$4 \times \pi \times (0.6\ \text{nm})^2 = 4.5\ \text{nm}^2 \qquad (1)$$

Area per PET molecule on a AuC surface was estimated to be 0.16 nm^2 ligand^{-1} according to the previous literature.[6] The length of PET was estimated by Avogadro 1.1.1 program. The MALDI-TOF MS and ICP-AES indicated that the chemical composition of PET/AuCs was $Au_{39}(PET)_{26}$. Therefore, Surface coverage of PET was,

$$26 \times 0.16\ \text{nm}^2 = 4.2\ \text{nm}^2 \qquad (2)$$

Using equation (1) and (2), the PET coverage was,

$$4.2\ \text{nm}^2 / 4.5\ \text{nm}^2 = 0.93 \qquad (3)$$

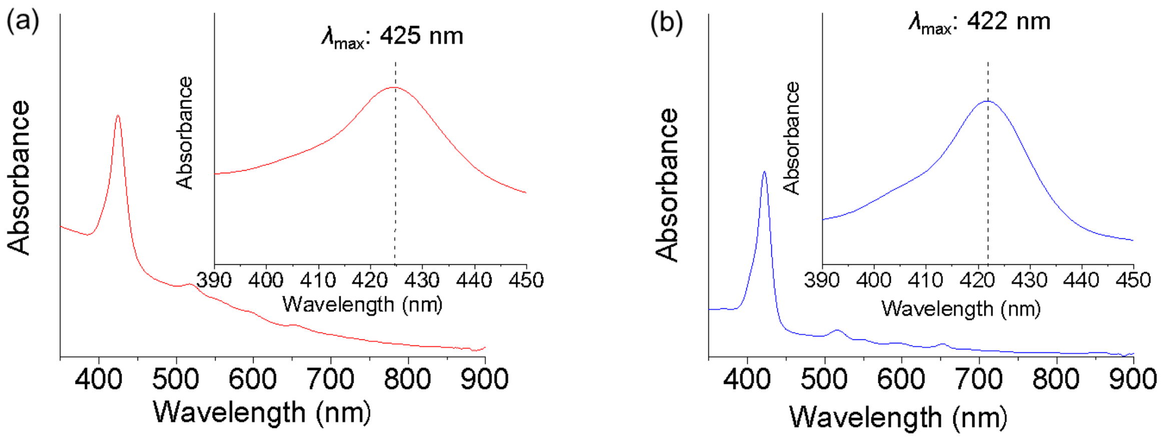

Figure S11. Absorption spectra of (a) SC_1P/AuCs and (b) SC_2P/AuCs after HER in DMF.

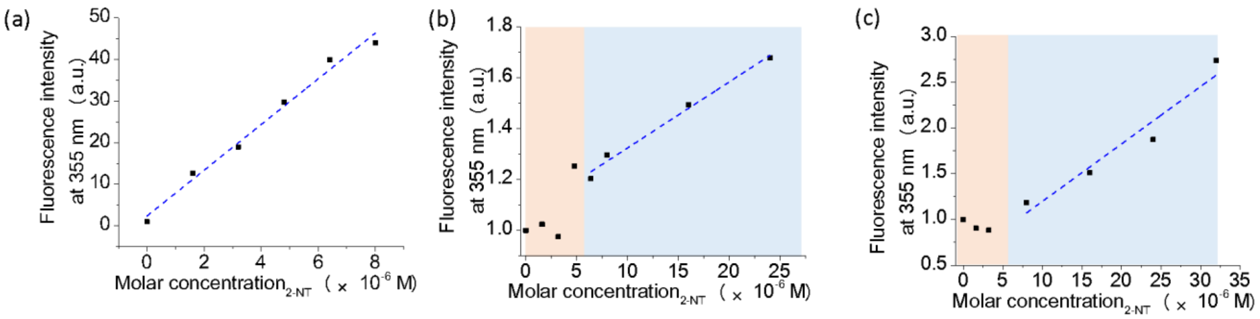

Figure S12. Plot of fluorescence intensity versus (a) molar concentration of 2-naphthalenethiol (2-NT), and the mixed solution of 2-NT and (b) SC_1P/AuCs (1.6 μM) and (c) SC_2P/AuCs (1.6 μM) excited at 283 nm in CH_2Cl_2 and DMF (1:1 (v/v)). The molecular accessibility was carried out based on previous work.[7] First, the 2-NT attached on the surface of AuCs resulted in quenching the fluorescence (red region). After saturation of adsorption sites on the Au surface, the free 2-NT showed fluorescence (blue region).

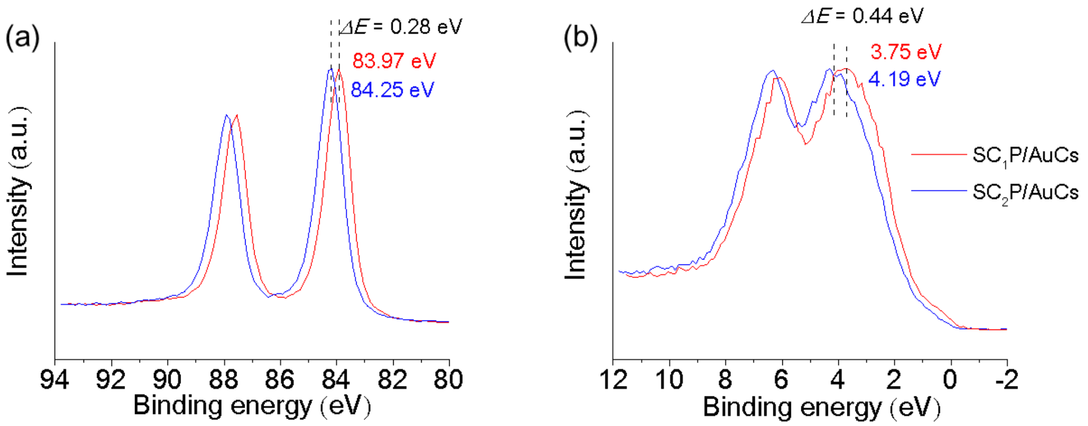

Figure S13. XPS spectra of SC_1P/AuCs (red) and SC_2P/AuCs (blue) in (a) Au4*f* and (b) Au5*d*. Binding energy differences between SC_1P/AuCs and SC_2P/AuCs of Au4$f_{7/2}$ and Au5$d_{5/2}$ were 0.28 and 0.44 eV, respectively. Notably, these differences were not derived from their charging up.

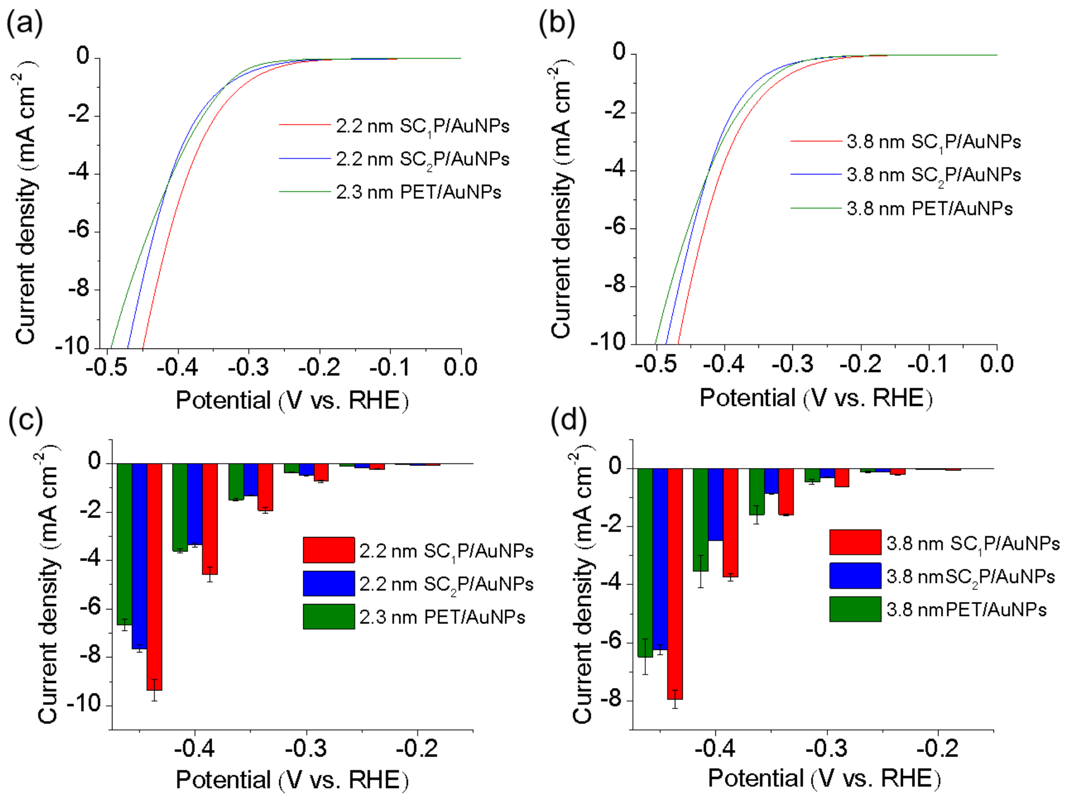

Figure S14. HER polarization curves for ligand-protected (a) 2.2-nm and (b) 3.8-nm AuNPs in 0.5 M phosphate buffer solution (pH 6.7). Comparison of current densities for ligand-protected (c) 2.2-nm and (d) 3.8-nm AuNPs at different potentials. Error bars indicate standard deviations from three runs with freshly deposited samples.

Table S2. Optical properties of SC_nP, SC_nP/AuCs, and SC_nP/AuNPs.

Compounds	λ_{max} (nm)	ε ($\times 10^5$ M^{-1} cm^{-1})	FWHM (nm)
SC_1P	420	3.9	13
SC_1P/AuCs	425	1.1	21
2.2 nm SC_1P/AuNPs	426	0.48	27
3.8 nm SC_1P/AuNPs	426	0.26	34
SC_2P	418	3.9	12
SC_2P/AuCs	422	2.1	19
2.2 nm SC_2P/AuNPs	422	0.65	19
3.8 nm SC_2P/AuNPs	424	0.56	18

Table S3. Summary of catalytic parameters for SC_nP/AuCs, SC_nP/AuNPs, PET/AuCs, and PET/AuNPs in the HER.

Compounds	Onset potential (mV)	Overpotential at 10 mA cm^{-2} (mV)	Current density at −0.4 V vs RHE (mA)	Tafel slope (mV dec^{-1})
SC_1P/AuCs	−390	470	−2.8	106
SC_2P/AuCs	−440	500	−0.84	88.6
2.2 nm SC_1P/AuNPs	−330	460	−4.8	101
2.2 nm SC_2P/AuNPs	−380	470	−3.4	105
3.8 nm SC_1P/AuNPs	−380	470	−3.7	114
3.8 nm SC_2P/AuNPs	−390	490	−2.5	111
PET/AuCs	−440	540	−0.61	101
2.3 nm PET/AuNPs	−370	490	−3.6	87.8
3.8 nm PET/AuNPs	−400	500	−3.5	96.6
7.3 nm PET/AuNPs	−420	510	−2.4	82.3
14.3 nm PET/AuNPs	−470	530	−1.7	91.3

References

[1] M. Sakamoto, D. Tanaka, H. Tsunoyama, T. Tsukuda, Y. Minagawa, Y. Majima and T. Teranishi, *J. Am. Chem. Soc.*, 2012, **134**, 816–819.

[2] C.-F. Chen, S.-D. Tzeng, H.-Y. Chen, K.-J. Lin and S. Gwo, *J. Am. Chem. Soc.*, 2008, **130**, 824–826.

[3] J. W. Slot and H. J. Geuze, *Eur. J. Cell Biol.*, 1985, **38**, 87.

[4] I. Ojea-Jiménez, L. García-Fernádez, J. Lorenzo and V. F. Puntes, *ACS Nano*, 2012, **6**, 7692–7702.

[5] X. Liu, M. Atwater, J. Wang and Q. Huo, *Colloids Surf. B*, 2007, **58**, 3–7.

[6] H. Hinterwirth, S. Kappel, T. Waitz, T. Prohaska, W. Lindner and M. Lämmerhofer, *ACS Nano*, 2013, **7**, 1129-1136.

[7] N. de Silva, J. M. Ha, A. Solovyov, M. M. Nigra, I. Ogino, S. W. Yeh, K. A. Durkin and A. Katz, *Nat. Chem.*, 2010, **2**, 1062-1068.
